# Dry season ecology of *Anopheles gambiae *complex mosquitoes in The Gambia

**DOI:** 10.1186/1475-2875-7-156

**Published:** 2008-08-18

**Authors:** Musa Jawara, Margaret Pinder, Chris J Drakeley, Davis C Nwakanma, Ebrima Jallow, Claus Bogh, Steve W Lindsay, David J Conway

**Affiliations:** 1 Medical Research Council Laboratories, Fajara, P.O. Box 273, Banjul, The Gambia; 2 Department of Infectious and Tropical Diseases, London School of Hygiene and Tropical Medicine, London, WC1E 7HT, UK; 3 The Sumba Foundation, P.O. Box 2148, Kuta 80361, Bali, Indonesia; 4 School of Biological and Biomedical Sciences, Durham University, Durham, DH1 3LE, UK

## Abstract

**Background:**

Malaria in The Gambia is highly seasonal, with transmission occurring as *Anopheles gambiae s.l*. populations expand during and immediately after a single annual rainy season that lasts from June to October. There has been very limited investigation of the ecology of vectors during the dry season, when numbers are very limited and distributions may be restricted.

**Methods:**

Weekly adult mosquito collections (pyrethrum spray, light trap, and search collections from rooms, as well as light trap collections from animal shelters, abandoned wells and grain stores), and artificial sentinel breeding site surveys were performed in four villages near the upper tidal and partially saline part of the Gambia River in the last four months of an annual dry season (March to June). Mosquito species were identified by morphological and DNA analysis, and ELISA assays were performed to test for *Plasmodium falciparum *sporozoites and human blood meal components.

**Results:**

Adults of *An. gambiae s.l*. were collected throughout the period, numbers increasing towards the end of the dry season when humidity was increasing. Adult collections were dominated by *An. melas *(86%), with *An. gambiae s.s*. (10%) and *An. arabiensis *(3%) also present throughout. Most females collected in room search and spray collections contained blood meals, but most from light traps were unfed. None of the females tested (n = 1709) contained sporozoites. Larvae (mostly *An. gambiae s.s*.) were recovered from artificial sentinel breeding sites in the two villages that had freshwater pools. These two villages had the highest proportions of *An. gambiae s.s*. adults, and experienced the most substantial increase in proportions of *An. gambiae s.s*. after the onset of rains.

**Conclusion:**

During the dry season population minimum, *An. melas *was the predominant vector species, but differences among villages in availability of fresh-water breeding sites correlate with egg laying activity and relative numbers of *An. gambiae s.s*. adults, and with the increase in this species immediately after the beginning of the rains. Local variation in dry season vector persistence is thus likely to influence spatial heterogeneity of transmission intensity in the early part of the rainy season.

## Background

Malaria transmission in The Gambia occurs mainly within a few months of each year, due to a single rainy season from June to October which creates breeding sites for three members of the *Anopheles gambiae *complex (*An. gambiae s.s*. and *An. arabiensis *that breed in fresh water, and *An. melas *that breeds in partially saline water around the tidal part of the River Gambia and its tributaries) [1]. The incidence of clinical cases and mortality peaks between September and November, and then rapidly declines [2]. A proportion of individuals retain asymptomatic infections during the dry season, and the small numbers of clinical cases of malaria seen during the middle and end of the dry season are considered to be mostly due to parasitological recrudesence. Some infected individuals carry gametocytes throughout the dry season, potentially allowing malaria transmission if vectors have the capacity during this period, and being the source of the transmission that occurs after the beginning of the rainy season [3].

Previous surveys of the *An. gambiae *complex in The Gambia and surrounding areas in Senegal have shown *An. gambiae s.s*. to be widely present, while its sibling species *An. arabiensis *is more common in inland areas, and *An. melas *is common in coastal and riverine areas that are tidal and close to mangrove forest [1]. Populations of *An. gambiae s.s*. are the most highly seasonal, whereas some suitable breeding sites for *An. melas *continue to exist during the dry season [4,5], and *An. arabiensis *tolerates the dry season better than does *An. gambiae s.s*. [6]. Immediately north of The Gambia, the village of Ndiop in Senegal is similar to most rural communities in The Gambia, where perennial breeding sites have not been identified, and highly seasonal transmission is due to *An. arabiensis *and *An. gambiae *s.s. [7]. In contrast, the nearby village of Dielmo is adjacent to a large freshwater breeding site that persists throughout the dry season, allowing *An. gambiae*, *An. arabiensis*, and *Anopheles funestus *to contribute to perennial transmission [8], a situation that has not been seen in any site in The Gambia. In surveys of Barkedji village in the drier Sahelian area of northern Senegal, *An. arabiensis *predominated and persisted longer than *An. gambiae *s.s. after the rains had terminated, but neither were found in the late dry season [9].

Although there appears to be virtually no transmission of malaria in most locations in The Gambia or Senegal during the dry season [6], it is important to study vector species ecology to know whether the annual population explosion could be reduced by interventions before or at the start of the rains. It is not known whether adults are widespread, or whether they survive only in a limited number of dry season refuge sites and then colonize adjacent areas from where they were seasonally absent. A study of *An. arabiensis *in the very long dry season in Sudan revealed that mosquitoes could be found mostly in local houses, and in the few areas where breeding sites persisted they could be reproductively active, but in more arid areas they rested awaiting immediate egg laying opportunities or underwent gonotrophic dissociation in which egg development was delayed until the rainy season [10]. The possibility that *Anopheles *eggs may survive in the dry soil of seasonal breeding sites has been investigated in western Kenya, by collecting soil samples and attempting to rear larvae in the laboratory [11]. This showed that eggs from field collected females of *An. gambiae *remain viable for approximately 12 days, suggesting that eggs on soil are not an effective life cycle stage to survive a long dry season. Therefore, a focus on the adult stage is warranted, with the possibility that identification and targeting of the dry season adult vector population could delay or reduce the seasonal increase after the rains.

To better understand the dry season ecology and transmission potential of the vector species in The Gambia during the driest period of the year, four villages were selected for intensive collections of adult mosquitoes as well as surveys of potential breeding sites and larvae developing in artificial breeding pans. The locations, relative densities and composition of the species, as well as reproductive parity, blood meal and sporozoite indices, were examined to investigate the conditions that may sustain the populations and influence their potential as vectors.

## Methods

### Study area

**Figure 1:**
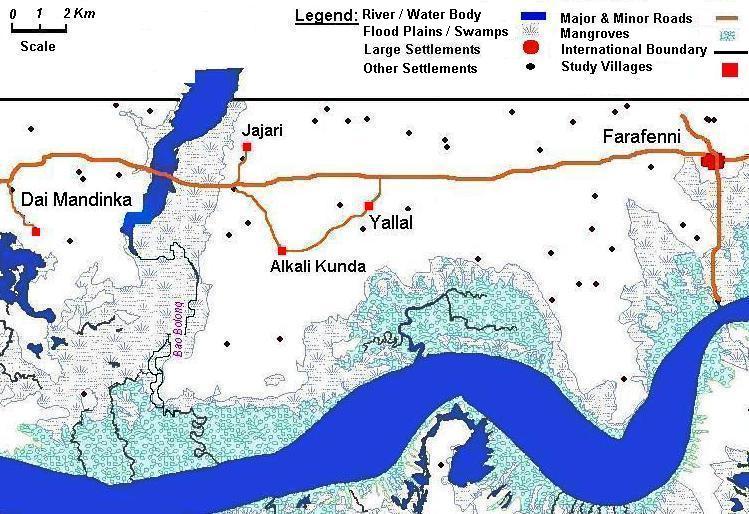
**Map of the study area in the North Bank District of The Gambia, showing the four study villages located west of Farafenni town.** The large water body at the bottom of the Figure is the Gambia River, and that at the top left surrounded by floodplains is the Bao Bolong tributary.

Mosquito sampling was conducted in the last 15 weeks of the dry season from March 13^th ^to June 21^st ^2000 in four villages located west of Farafenni town in the North Bank Division of The Gambia (Figure 1). The last week of sampling followed the first rainfall (9.5 mm on June 13^th^) but was considered as a dry season sample of adult mosquitoes as these could not have emerged from rain-fed breeding sites (a minimum of 8 days is needed for development from eggs to adults). The villages were Yallal (population of 495 in 52 compounds), Alkali Kunda (abbreviated here as Alkali, population of 889 in 58 compounds), Jajari (population of 752 in 32 compounds), and Dai Mandinka (abbreviated here as Dai, population of 246 in 9 compounds). Two of the villages (Dai and Jajari) are less than 1 km from the Bao Bolong (a tributary of river Gambia with marshland along its banks that remain flooded for most of the dry season). The other two are over 4 km away from the Bao Bolong, and all are several kilometres away from the main River Gambia that is mostly bordered by *Rhizophora *sp. and *Avicennia *sp. mangrove in this tidal region approximately 100 km upstream from the river mouth. Data on maximum and minimum daily temperature and humidity were collected at the government meteorological station at Yallal village, which is approximately 3 km from Alkali, 6 km from Jajari and 10 km from Dai.

Prior to the study, permission was sought from the village elders, following which village meetings were conducted to explain the purpose of the study, and participation requested. Verbal consent from house owners and their compound heads for permission to collect mosquitoes from their houses followed this. Ethical approval was given by the MRC and Gambian Government Joint Ethics Committee.

### Sampling of adult mosquitoes

Pyrethrum spray collections (PSC) were conducted weekly in five randomly selected rooms in each of the four villages. Collections were performed in rooms that had not used any form of insecticide or repellent during the previous week. The average number of people sleeping in a room ranged from 1–10 (with a mean of 3). Foodstuff and utensils were removed from the room and a white sheet of cloth spread over the floor and furniture. Insecticide aerosol containing Tetramethrin 0.10%, d-Allethrin 0.10%, Dichlorovos 0.05% and Permethrin 0.02% from a pressurized can (trade mark "BOP") was sprayed inside the room for 5–10 seconds and left with the doors and windows closed for 10 minutes, after which all the dead and immobilized mosquitoes were collected from the white sheet into a labelled cup.

Light trap collections (LTC) were performed by setting seven CDC light traps weekly in four different types of locations in each village (houses, abandoned wells, animal shelters and grain stores). The first four light traps were located in randomly selected homes in each village and the remaining three assigned between the other types of locations (sampling of these was not randomized but depended primarily on accessibility and availability).

Room search collections (RSC) were performed by randomly selecting ten rooms in each village each week to be searched for mosquitoes. In each room, a 20 minute search was conducted using a pooter tube, torchlight and a ladder. Searches were made around the room including underneath bed nets, around drinking jars, hanging clothes, cracks and crevices in the wall, and ceilings.

### Sampling of mosquito larvae

An area of radius 1 km from the edge of each village was searched for any permanent water bodies. Those found were recorded and inspected for mosquito eggs or larvae, during most weeks of the survey period. Artificial breeding pans were used to survey for new egg laying. Sixteen open metal bowls (60 cm diameter and 18 cm in depth) were sunk into the ground, with the rim at ground level, and filled with fresh water, one in each quarter of each of the four villages, in attempt to attract egg laying mosquitoes. Bowls were inspected twice a week for eggs and larvae and topped with water drawn from the village well or bore hole. Once a week the water was emptied through a sieve (1 mm pore size) to collect all larvae or eggs, and refilled with fresh water. Larvae collected were transported to an insectary at Farafenni to be reared to adult stage for identification.

### Mosquito sample processing and analysis

Counts of adult anopheline and culicine mosquitoes were recorded in each collection, and female anopheline mosquitoes examined further. Blood fed mosquitoes had their blood meal squashed on Whatman no 1 filter paper, which was then stored dry with a desiccant at room temperature until testing for blood meal analysis. Blood components from the spots were eluted in normal saline overnight and the blood meal tested for human components by an ELISA assay [12]. Legs and wings of individual *An. gambiae s.l*. mosquitoes were kept in eppendorf tubes with silica gel desiccant for later molecular identification of sibling species, and analysis of *An. gambiae s.s*. molecular forms (M and S) in a subset of individuals, using a PCR-RFLP method previously described [13]. The head and thorax of each dissected mosquito was kept in a separate well of a 96 well microtitre plate, for later analysis of *P. falciparum *sporozoite infection using ELISA [14]. Tests for differences in proportions of categorical variables, including species and blood meal indices, were performed by chisquare analysis.

## Results

### Dry season collections of adult mosquitoes

**Figure 2:**
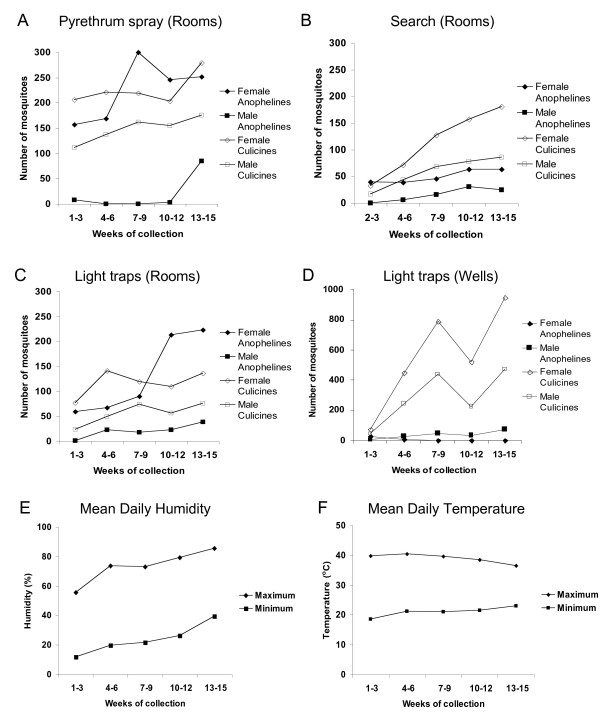
**Numbers of adult mosquitoes collected in each of the four major collection types in 3 week periods throughout the 15 weeks at the end of the dry season (March to June) in 2000 (overall numbers of collections of each type are given in Table 1)**. A, Pyrethrum spray collections in rooms; B, Room search collections (these began in week 2); C, Light trap collections in rooms; D, Light trap collections in wells; E, Mean daily humidity maximum and minimum; F, Mean daily temperature maximum and minimum.

Figure 2 shows the numbers of female and male anophelines and culicines collected in three week periods over the 15 weeks until the end of the dry season, with each of the four main methods (pyrethrum spray, search, and light trap collections from rooms, and light trap collections from wells). With most collection methods, the numbers of all mosquitoes increased towards the end of the dry season. Culicines were more abundant in most of the collections throughout, except for pyrethrum spray and light trap collections from rooms, in which female anophelines were most common towards the end of the dry season (Figure 2A–D). Although there was no rain, relative humidity increased over this time (a normal trend towards the end of the annual dry season) (Figure 2E), while maximum and minimum daily temperature patterns remained more constant (Figure 2F).

### Location of females of the *Anopheles gambiae *complex

Almost all anophelines collected belonged to the *An. gambiae sensu lato *(*s.l*.) complex. The numbers of *An. gambiae s.l*. per collection for each of the six collection types are shown in Table 1. A total of 300 pyrethrum spray collections were conducted over the whole 15 week period, from five rooms each week in each of the four villages. Most (72%) of the collections yielded *An. gambiae s.l*. mosquitoes, with a total of 1,125 females collected (mean of 3.8 per room collection). Room searches commenced in the second week and were conducted for 14 weeks, during which 560 searches were conducted, of which 23% yielded anopheline mosquitoes, with a total of 248 female *An. gambiae s.l*. (mean of 0.4 per room collection). *Anopheles gambiae s.l*. mosquitoes were found in virtually in all parts of the rooms, but were particularly found in and around water jars, on the wall and in cracks in the wall, in and outside the bed net, on the ceiling and roof fringes and from hanging clothes.

**Table 1:** Summary of March – June 2000 collections with examinations of *An. gambiae s.l*. females for reproductive status, blood meal and sporozoite indices

	Collection type:					
	Spray (Rooms)	Search (Rooms)	Light trap (Rooms)	Light trap (Wells)	Light trap (Animal shelters)	Light trap (Grain stores)
No. of collections	300	560	235	101	54	11
No. of *An. gambiae* * s.l*. females	1125	248	652	50	46	20
*An. gambiae* * s.l*. females per collection	3.75	0.44	2.77	0.49	0.85	1.82
Proportion parous (%)	93	95	93	88	88	100
Proportion gravid (%)	12	10	23	4	6	5
Proportion with blood meal (%)	83	82	27	22	20	25
No. positive for sporozoites	0/1104	0/229	0/332	0/15	0/17	0/12

Over the 15 week period, 235 light trap collections were performed in houses occupied by people sleeping under bednets, 39% of which yielded *An. gambiae s.l*. females with a total of 652 (mean of 2.8 per room collection). In addition, 17 *An. rufipes *individuals were identified overall. Out of 101 light trap collections in abandoned wells, a total of 50 *An. gambiae s.l*. females were recovered (mean of 0.5 per well collection). An average of 7 traps was set per week over the 15 weeks, and the highest number of *An. gambiae s.l*. (28) was collected in the first week, whereas the rest of the catches had a range of 0–8 per week. Only 54 light trap collections were conducted in animal shelters, yielding 46 *An. gambiae s.l*. (mean of 0.9 per shelter collection). Due to lack of access, only 11 light trap collections were set in grain stores (over 7 weeks commencing with week 3), yielding 20 *An. gambiae s.l*. (mean of 1.8 per grain store collection).

Most female *An. gambiae s.l*. that were resting in houses and collected by room search or pyrethroid spray methods contained blood meals (Table 1), whereas most of those collected by light traps (whether in rooms, wells, animal shelters, or grain stores) were unfed. Of the fed mosquitoes, 35% (566 of 1597 tested by ELISA) contained human blood. The proportion containing human blood was higher in room search collections (73%) than with other collection methods including pyrethrum spray collections from rooms (31%) (P < 0.001). None of 1709 *An. gambiae s.l*. females tested contained sporozoites.

### Molecular identification of *Anopheles gambiae *complex species

**Figure 3:**
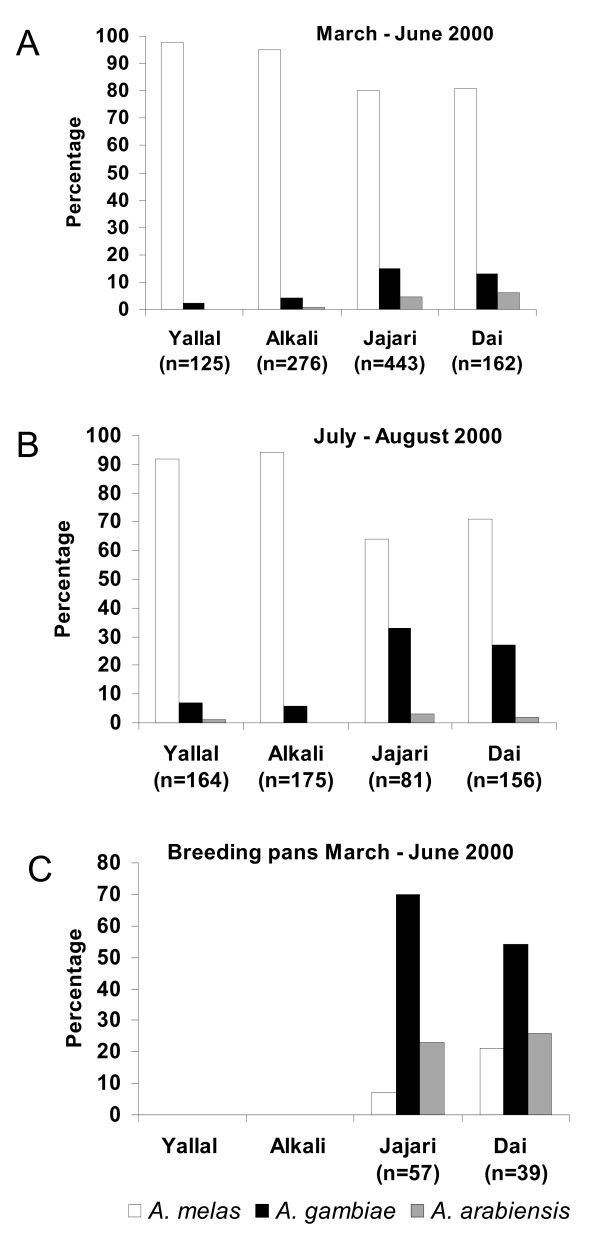
**Proportions of each of the 3 local sibling species of *An. gambiae s.l*.** in a random sub-sample tested from all adult collections from the four study villages. A, during the late dry season (March – June 2000); B, during the early rainy season (July – August 2000); C. in artificial breeding pans placed in each of the villages during the late dry season (March – June 2000).

A total of 1,002 adult *An. gambiae s.l*. (from the pyrethrum spray catches, room search collections and light trap catches) for all the four villages between March and June were identified to species level by PCR. The results showed 865 (86%) to be *An. melas*, 103 (10%) *An. gambiae s.s*. and 34 (3%) *An. arabiensis*. A random sample of 47 *An. gambiae s.s*. mosquitoes were typed for X-chromosomal molecular form, showing 34 (72%) to be S form and 13 (28%) to be M form. The proportions of *An. gambiae s.s*. and *An. arabiensis *in Jajari and Dai were higher than in the other two villages (Figure 3A). A random sample of 576 *An. gambiae s.l*. from weekly room collections during six weeks in July and early August (following the start of the rainy season) was identified to species by PCR, showing a significant increase in the proportion of *An. gambiae s.s*. compared to the other species, particularly in Jajari and Dai (P < 0.01 for each, Figure 3B).

### Dry season collections and species identification of mosquito larvae

There were eight observed shallow wells or pools maintained in communal vegetable gardens around Jajari village, used by the women used for watering plants during the dry season, and also a few isolated pools used for cattle drinking around Dai (three pools) and Jajari (two pools). There were 56 inspections of permanent water bodies and five inspections of temporary water bodies that occasionally were seen around wells, none of which revealed any mosquito larvae. No persistent pools of water were seen in the other two villages.

Mosquito larvae were seen only in the artificial breeding pans, in 28% (133) of 475 inspections from all villages. Altogether, 7,274 larvae (including culicines) were collected and transported alive to an insectary and 5,617 of these reared to adults and identified by morphology. There were 374 reared anophelines (all *An. gambiae s.l*.) from two of the villages (Jajari and Dai), but none from the other two villages. PCR analysis of a random sample of these reared *An. gambiae s.l*. from Jajari and Dai showed that most (70% and 54% respectively) were *An. gambiae s.s*. (Figure 3C).

## Discussion

Adults of three members of the *An. gambiae *complex (mostly *An. melas*, but with *An. gambiae *and *An. arabiensis *occurring alongside) were present in low numbers throughout the last three months of the dry season in each of the four study villages. Most were found inside houses, with the pyrethrum spray and light trap methods yielding the highest numbers per collection. Room search collections indicated common resting sites were around water jars, where it is humid and cool, on the walls, hanging clothes, cracks and crevices in the wall, and the edges of the roof. Over 80% of those collected by pyrethrum spray and room search methods contained blood meals which is similar to the proportion found during the wet season [15]. A study of *An. arabiensis *in the dry season of Sudan [16] indicated that most dry season adults were in houses, with smaller numbers in wells or animal burrows. Consistent with this, the density in light trap collections from wells, animal shelters and grain stores here was lower than from inhabited rooms, although it should be noted that light traps sample outdoor populations of mosquitoes less efficiently than indoor populations [17]. The presence of male *An. gambiae s.l*. in almost all the collections suggests that breeding may continue throughout the dry season. Both males and females were more abundant in collections towards the end of the dry season, concurrent with a humidity increase, similar to previous findings in northern Nigeria [18] suggesting that reproductive activity may increase in anticipation of available egg laying sites. Although an earlier study in Sudan showed that some females entered a dry season aestivation state, collections there also included males and blood fed females, and indicated that breeding occurred wherever possible [10].

No anopheline larvae were collected from the permanent water bodies in or around the villages. The artificial breeding pans in two of the villages (Jajari and Dai) were colonized by each of the three local species of *An. gambiae s.l*. (dominated by *An. gambiae s.s*.), confirming that mosquitoes in these villages were reproductively active. These two villages are closer to the Bao Bolong tributary with plains that have occasional isolated pools where larval development could occur, so it is possible that ongoing breeding occurs somewhere within permanent sites that were not identified for sampling. The other two villages were further away from any potential breeding site and the artificial breeding pans in these villages were not colonized by any anophelines (only culicine larvae were collected in breeding pans in these villages), so it is possible that the gonotrophic cycle is arrested in these areas or that eggs produced are retained for laying at the beginning of the rainy season. Overall, more than 90% of the female *An. gambiae s.l*. collected were parous, indicating that most individuals are old (and that relatively few new adults emerge during the dry season). This is much higher than a 53% parity estimated during a previous wet season survey nearby in the North Bank District [15], when many mosquitoes would be expected to be recently emerged. It is also in marked contrast to a parity of 45% found in an area of irrigated rice production further upriver in the Central River District during a previous dry season [6], which is likely to reflect different ecology. In the present study there were few active breeding sites, whereas in the irrigated rice production area breeding sites were abundant in the dry season and there would thus be many younger mosquitoes.

The flooded alluvial plains that are on the edge of mangrove swamps are the primary breeding areas of *An. melas *[4,19]. The landward edge of flooded alluvial plains was previously identified as the main breeding area of *An. gambiae s.l*. (mostly *An. melas*) in transects of several nearby sites around the tidal part of the Gambia river during the rainy season [20]. These pools shrink during the dry season, but many do not disappear, which allows breeding of *An. melas *to continue throughout the dry season [19]. Breeding of *An. gambiae s.s*. and *An. arabiensis *is strictly limited to sites of low salinity, and these are generally rare in the dry season which would explain why these species were more common in the two villages that had permanent pools for watering plants and animals. These villages also had larvae detected by the sentinel breeding pans, whereas villages without permanent pools did not, indicating that adults of these vector species are reproductively active only within very localized areas. The significant increase in the proportion of *An. gambiae s.s*. in these two villages after the beginning of the rains suggests the importance of the localized dry season populations.

It is not practically possible to estimate population sizes of each of the species during the dry season, as numbers are too low to allow a mark-release-recapture approach. Recognizing this, two previous studies in Mali [21] and Senegal [22], applied a population genetic analysis of microsatellite allele frequencies to estimate the genetically effective population size of *An. arabiensis *in particular study villages. Each of these studies indicated that the genetically effective population size was maintained above a substantial level (at least ~10^3 ^and possibly much higher) so that there was no genetic bottleneck, even though hardly any mosquitoes were found during the dry season. The present study identifies the dry season location and breeding of *An. arabiensis*, although this was the least common of the three sibling species, so it is unlikely that any of the species undergoes a seasonal genetic bottleneck in The Gambia.

Although dry season vector population sizes are not as low as previously supposed, and blood feeding on humans as well as animals continues, dry season transmission of *Plasmodium falciparum *in the study area is unlikely as none of 1,709 mosquitoes examined had sporozoites, a lower prevalence than in reported wet season surveys in The Gambia (in which most sporozoite prevalences are 0.2–3.0%) [23]. A previous year-round study in a village within an irrigated rice production area in the Central River District showed very seasonal sporozoite prevalence in *An. gambiae s.l*. (*An. gambiae s.s*. and *An. arabiensis*; *An. melas *being absent due to the absence of salinity in that area), being virtually zero in the dry season [6]. Elsewhere in coastal areas of West Africa, *An. melas *may maintain transmission during the dry season, as indicated by a survey in Lagos where there is a shorter dry season [24]. It is worth noting that most of the *An. gambiae s.s*. typed in the present study had small subunit rRNA molecular form S (73%), whereas typing of samples from a small number of other areas in The Gambia previously showed nearly all to have the alternative M form [25]. There is a need to more fully understand the population genetic structure and distribution of each of the vector species in The Gambia, and update the only systematic survey data that were collected more than 25 years ago [1].

It is likely that rainy season population sizes and malaria transmission intensities within villages are influenced to some extent by local dry season populations, although this may be more pronounced in the early rainy season than later on as a mark-release-recapture experiment previously conducted showed that some *An. gambiae s.l*. dispersal can occur between adjacent villages [26] and there is evidence for occasional long-distance flight by mosquitoes [27]. Although interventions are not needed to interrupt transmission in the dry season, approaches to reduce vector populations might be effectively targeted at this time. For example, application of indoor residual spraying shortly before the annual rains would have remove a large proportion of the adult vector population that rests within houses in the dry season, and thus may delay the start of the transmission season (as well as having an ongoing residual effect during the transmission season). Studies on the effectiveness of annual indoor residual spraying and other household based interventions at the late stage of the dry season would now be important, with comparison among villages that have different proportions of vector species.

## Conclusion

During the dry season, in villages near the tidal part of the Gambia River, *An. gambiae s.l*. mosquitoes are at an annual population minimum, but this study showed male and female adults to be present in houses and other buildings and to be collectable by several methods that allowed for comparisons over space and time. Numbers collected increased towards the end of the dry season as humidity increased. Most females in room search and spray collections contained blood meals, but most from light traps were unfed, and none contained sporozoites. *An. melas *was the predominant species, but differences among villages in availability of fresh-water breeding sites correlated with egg laying activity and relative numbers of *An. gambiae s.s*. adults, and with the increase in this species immediately after the beginning of the annual rains. This local variation in dry season vector persistence is likely to influence the spatially variable transmission intensity among communities during the rainy season, and could be evaluated further as a potential means of targeting control.

## Competing interests

The authors declare that they have no competing interests.

## Authors' contributions

MJ, MP, CJD, CB, and SWL designed the field sampling and the initial study outline. MJ and EJ conducted the entomological collections and most laboratory analyses. DN and DJC supervised and checked the molecular species and M and S form typing. MJ, MP and DJC performed the data analyses. MJ and DJC wrote the paper, incorporating comments and suggestions from all authors. All authors read and approved the final manuscript.
